# Elevated p16ink4a Expression in Human Labial Salivary Glands as a Potential Correlate of Cognitive Aging in Late Midlife

**DOI:** 10.1371/journal.pone.0152612

**Published:** 2016-03-30

**Authors:** Christiane Elisabeth Sørensen, Katerina Tritsaris, Jesper Reibel, Martin Lauritzen, Erik Lykke Mortensen, Merete Osler, Anne Marie Lynge Pedersen

**Affiliations:** 1 Section of Oral Medicine, Clinical Oral Physiology, Oral Pathology and Anatomy, Department of Odontology, Faculty of Health and Medical Sciences, University of Copenhagen, Copenhagen, Denmark; 2 Department of Cellular and Molecular Medicine, Faculty of Health and Medical Sciences, University of Copenhagen, Copenhagen, Denmark; 3 Center for Healthy Aging, Faculty of Health and Medical Sciences, University of Copenhagen, Copenhagen, Denmark; 4 Department of Neuroscience and Pharmacology, Faculty of Health and Medical Sciences, University of Copenhagen, Copenhagen, Denmark, and Department of Clinical Neurophysiology, Rigshospitalet-Glostrup, Glostrup, Denmark; 5 Department of Public Health, Faculty of Health and Medical Sciences, University of Copenhagen, Copenhagen, Denmark; 6 Danish Aging Research Center, Universities of Aarhus, Southern Denmark and Copenhagen, Odense, Denmark; 7 Research Center for Prevention and Health, Rigshospitalet-Glostrup, Glostrup, Denmark; National Institutes of Health, UNITED STATES

## Abstract

**Background:**

The cell-cycle inhibitor and tumor suppressor cyclin dependent kinase inhibitor, p16^ink4a^, is one of the two gene products of the ink4a/ARF (cdkn2a) locus on chromosome 9q21. Up-regulation of p16^ink4a^ has been linked to cellular senescence, and findings from studies on different mammalian tissues suggest that p16^ink4a^ may be a biomarker of organismal versus chronological age.

**Objective:**

The aim of this study was to examine the immunolocalization pattern of p16^ink4a^ in human labial salivary gland (LSG) tissue, and to analyze whether its expression level in LSGs is a peripheral correlate of cognitive decline in late midlife.

**Methods:**

The present study was a part of a study of causes and predictors of cognitive decline in middle-aged men in a Danish birth cohort. It is based on data from 181 male participants from the Danish Metropolit birth cohort, born in 1953, who were examined for age-associated alterations in cognition, dental health, and morphological and autonomic innervation characteristics of the LSGs. The participants were allocated to two groups based on the relative change in cognitive performance from young adulthood to late midlife. LSG biopsies were analyzed by qRT-PCR for the expression level of p16^ink4a^. Immunohistochemistry was performed on formalin-fixed, paraffin-embedded sections of LSGs.

**Results:**

p16^ink4a^ immunoreactivity was observed in LSG ductal, myoepithelial, and stromal cells, but not in acinar cells. The mean relative expression of p16^ink4a^ in LSGs was higher in the group of participants with decline in cognitive performance. A logistic regression analysis revealed that the relative p16 expression was predictive of the participant’s group assignment. A negative correlation was found between relative p16^ink4a^ expression and the participant’s standardized regression residuals from early adulthood to late midlife cognitive performance scores.

**Conclusions:**

p16^ink4a^ expression in human LSGs may constitute a potential peripheral correlate of cognitive decline. Human labial salivary glands seem suitable for studies on organismal as opposed to chronological age.

## Introduction

Cognitive decline which exceeds the normal range of age-related cognitive changes may signal underlying pathological conditions. The earliest detection of such alterations is essential as it improves the chances to intervene and delay the potential progression into dementia. Emerging central nervous system alterations that lead to cognitive decline are difficult to detect. Thus, identification of potential correlates of abnormal cognitive aging is needed.

Organismal aging may partly result from a decline in regenerative processes as homeostatic tissue proliferation decreases with age. One mechanism, which is thought to play a significant role in diminishing tissue regeneration, is cellular senescence, otherwise known for its role in tumorigenesis prevention [1]. Cellular senescence is a distinct kind of “terminal differentiation” [2], which causes basically permanent growth arrest of mitosis-competent cells in response to different stressors, as for example oncogenic stimuli, telomere dysfunction or DNA damage [1]. Many senescent cells withstand apoptosis and accumulate in tissues with regenerative capacity [3–5]. The linkage between cellular senescence and aging may arise from the loss of mitosis-active cells in self-renewing tissues, and from alterations of the gene expression and secretory profiles of senescent cells, which develop a senescence-associated secretory phenotype (SASP) [6]. Thus, altered release of auto- and paracrine signaling molecules by senescent cells, potentially influences cellular development as well as structural and likely functional aspects of the tissue they reside within [6]. Though cellular senescence has mainly been described in cultured cells and peripheral tissues with regenerative potential, it may also play a role in normal and pathological aging processes within the central nervous system, since the brain harbors cells of non-neuronal origin with proliferative capacity (reviewed by [7]). Alteration of their secretory phenotype may contribute to neuroinflammatory processes, which could be causally involved in the etiology of age-related neurodegenerative disorders [7].

Cellular senescence can be induced by two different tumor suppressor pathways that are separately regulated by the two gene products p16^ink4a^ (in the following referred to as p16) and p14^ARF^ (p19^ARF^ in mice), encoded by the ink4a/Arf (also known as CDKN2A) locus on chromosome 9p21 [2]. p14^ARF^ mediates growth arrest by stabilizing the tumor suppressor p53 [8], whereas p16 induces cellular senescence by preventing the inactivation of the retinoblastoma protein (pRB) by the cyclin dependent kinases cdk4 and cdk6 [9,10], a step otherwise required for cell cycle progression. Mutations or deletions at the Ink4a/ARF locus have been observed in different human cancers [11]. On the other hand, the up-regulation of its expression has been suggested to represent a biomarker of organismal as opposed to chronological aging [12], since studies on human and animal tissues have shown that p16 expression enhances with age, e.g. [12–19].

Salivary glands are well-suited for the study of organismal aging and cellular senescence in relation to cognitive decline, since these organs on one side undergo distinct age-related structural changes and on the other hand possess the ability to regenerate. In normal human salivary glands the different parenchymal cell populations are maintained by proliferation of all parenchymal cell types, including myoepithelial, acinar and intercalated duct cells, oxyphilic cells of the intra- and interlobular ducts as well as basal cells [20]. While luminal acinar cells and cells of the intercalated ducts undergo independent proliferation, oxyphilic cells of the intra- and interlobular ducts are to the largest extent maintained by basal cells, which likely function as kind of reserve cells that are able to proliferate and differentiate [20]. Apart from the proliferation of differentiated parenchymal cells, it has also been investigated whether salivary glands possess adult stem cells which might be involved in glandular regeneration (reviewed by [21] and [22]).

The aim of the present study was to determine the localization of p16 in human labial salivary glands, and to analyze whether p16 expression levels represented a potential peripheral correlate of cognitive decline in late midlife.

## Methods

### Study participants

The expression analysis of p16 was performed on labial salivary gland tissue samples taken from the same group of middle-aged men, who participated in a clinical dental examination as part of a search for potential non-brain correlates for cognitive decline [23,24]. The detailed participant selection procedure is described elsewhere [23,25]. Briefly, participants were all males, born in 1953 and recruited from the Metropolit Cohort of the Copenhagen Aging and Midlife Biobank (CAMB) [26]. Cognitive function was followed longitudinally by two previous cognitive assessments. The first test, the Børge Priens Prøve (BPP) [27], was administered as part of the Danish military draft board evaluation, approximately at age 18 [25]. The second test was the Intelligenz-Struktur-Test 2000-R (I-S-T 2000 R) [28], administered as part of the CAMB data collection, approximately at age 56 [29]. Both tests comprised verbal analogies and number series subtests [25,29]. The correlation between the two tests was 0.70 after a retest interval of almost 40 years, and a regression model was used to predict the expected I-S-T 2000 R scores in late midlife from the earlier BPP test scores [25]. The largest negative or positive standardized residuals between the predicted and attained scores on the I-S-T 2000 R test were utilized to select one group with relatively increased (group 1) and a second group with relative decline in cognitive performance in late midlife (group 2). Men with major diagnosed psychiatric or neurodegenerative diseases, major structural brain lesions or drug and/or alcohol abuse were excluded. A total of 195 men participated in the clinical dental examination as previously described [24]. Out of these, 181 participants were included in the present study. The remaining 14 out of the 195 participants were not included due to the following reasons: Dropout from the study, not fulfilling inclusion criteria, not consenting to labial salivary gland biopsy, standard deviation (SD) of the qRT-PCR data triplicates exceeding the set limit value of 0.40, samples without successful qRT-PCR reaction and no RNA available for analysis. The study includes the use of human tissue samples, which was approved by the regional scientific ethical committee, the Capital Region of Denmark, no. H-3-2010-016. The participants were both informed by letter and orally and gave written consent.

### Labial salivary gland biopsy

Biopsies of labial salivary glands were taken under regional anesthesia from the lower lip as earlier described [24,30]. The glands were transferred to RNA*later*^®^ Stabilization Reagent (Qiagen) and stored at -80°C until analysis. Sections of labial salivary gland tissue used for the p16 immunohistochemistry were taken from the same biopsies that were used for the previously published histological analyses [24]. These samples had been fixed in 10% neutral buffered formalin (pH 7.3, 24 h, 20°C), embedded in paraffin and routinely processed [24].

### RNA isolation and quantitative real time PCR (qRT-PCR)

For total RNA isolation, the LSGs were transferred into lysis buffer (RNeasy^®^ micro kit, Qiagen) and homogenized by bead milling with 5 micron steel beads (Qiagen). Total RNA was purified from homogenized samples by use of the RNeasy^®^ micro kit (Qiagen), according to the manufacturer’s protocol. cDNA was synthesized by reverse transcription from 0.5 μg of total purified RNA, using Omniscript RT kit (Qiagen) and oligo(dT)primers (First-strand synthesis kit, Novagen), following the manufacturer’s instructions. The expression level of the CDKN2A gene, encoding p16, was determined by quantitative real-time PCR, with the following primers: forward (200 nM) 5’-ACG CCC TAA GCG CAC ATT C-3’ and reverse (300 nM) 5’-AGT GTG ACT CAA GAG AAG CCA G-3’. LightCycler^®^ 480 DNA SYBR Green I Master (Roche) was added to the samples and analysis was carried out with a Mx3005P thermal cycler (Agilent Technologies). The initial melting was performed at 95°C, 10 min. During the subsequent 40 amplification cycles, the melting temperature was 95°C (20s), the primer annealing temperature was 60°C (22s) and the elongation temperature was 72°C (20s). After completed amplification, melting curves were generated to confirm amplification specificity and standard curves were generated to ascertain amplification efficiency. Primer efficiencies were 95.9% for p16 and 97.1% for GAPDH, respectively. All samples were analyzed in triplicates (S1 Table). In the case of two participants triplicates of p16 levels were determined twice, and for another two participants p16 levels in two different RNA preparations were analyzed in triplicates. In these cases, the measurement with the lowest SD of the triplicates was included in the analysis. p16 Ct values were analyzed taking primer efficiency into account, and they were normalized to GAPDH and converted to relative p16 expression with GenEx, Multid Analyses AB Software.

### Immunolocalization of p16 in labial salivary gland tissue

Selection of samples used for p16 immunohistochemistry was based on relatively high (n = 3) and low (n = 5) p16 mRNA expression levels in the LSGs. The fixation of the biopsies and pretreatment procedure for immunohistochemistry has been described earlier [24]. Approximately 2 μm thin sections of paraffin-embedded labial salivary glands were deparaffinized in Tissue-Tek^®^ Tissue-Clear^®^ (Sakura Finetek Europe) and rehydrated in a series of descending alcohols. For antigen retrieval, the sections were transferred to Tris-EDTA-buffer, pH 9.0, and pretreated by microwave irradiation at 100°C (Samsung, 5 min at 600 W and 15 min at 300 W). Immunostaining was performed with the Dako REAL™ EnVision™ Detection System, Peroxidase/DAB+, Rabbit/Mouse kit (K5007, Dako, Denmark), following the manufacturer’s manual. After blocking of endogenous peroxidase (S2023, Dako, Denmark), the sections were rinsed and incubated with a primary antibody against p16 (p16 Clone JC8, SantaCruz, USA), diluted 1:100 in antibody diluent (S2022, Dako, Denmark). The incubation lasted 30 min at room temperature. The sections were then rinsed and incubated with secondary antibody conjugated with horseradish peroxidase labeled polymer for 30 min at room temperature. Substrate-chromogen 3,3’-diaminobenzidine (DAB, diluted 1:50) was utilized for antigen visualization, and Carazzi’s hematoxylin (Ampliqon, Denmark) was used for counterstaining of the sections. Sections were mounted in Pertex^®^ mounting medium (HistoLab, Sweden).

### Statistical analysis

The significance of group differences within the relative p16 expression, on the BPP and I-S-T 2000 R cognitive scores was tested by an unpaired t-test. The Wilcoxon Two Sample Rank Sum test was used for calculation of P-values for group differences with regard to the number of years of education, package years, and alcoholic drinks per week. P-values for group differences within smoking habits and daily alcohol consumption were obtained by Fisher’s exact or Chi-Square tests. These analyses were performed with GraphPad Prism version 6.0, GraphPad Software, San Diego, California, USA. A logistic regression model was used to predict group assignment from relative p16 expression levels. The correlation between the standardized regression residuals and relative p16 expression levels was estimated with Pearson’s correlation coefficient. These analyses were performed in STATA (Version 13), STATA Corporation, Texas, USA. The statistical tests were two-sided, and P<0.05 was considered statistically significant.

## Results

### Demographic data and cognitive performance scores

Participants with positive standardized regression residuals from BPP to I-S-T 2000 R, indicative of increased cognitive performance in late midlife, were allocated to group 1. Participants with negative standardized regression residuals, indicative of decline in cognitive performance with age, were assigned to group 2.

The participants had a mean (standard deviation (SD)) age of 57.8 (0.6) in group 1 (n = 87) and 58.0 (0.8) years in group 2 (n = 94). The two groups differed with respect to education as the mean number of years of education (SD) was 14.1 (2.3) years in group 1 (n = 87) and 12.6 (2.2) in group 2 (n = 94), (P<0.0001). Smoking habits did not differ significantly between the two groups, including the proportions of those who had never smoked (P = 0.27, n = 180), those who smoked daily (P>0.99, n = 180) or occasionally (P = 0.11, n = 180), those who had stopped smoking within one year (P = 0.20, n = 180) and more than one year (P>0.99, n = 180) before the examination. The mean number of package years was not significantly different between the two groups (P = 0.80, data available from 165 participants). Neither the number of persons who reported on daily alcohol consumption (P = 0.079, n = 181) nor the number of weekly consumed alcoholic drinks (P = 0.55, n = 181) varied significantly between the two groups. No significant group difference was found with respect to the BPP cognitive performance scores in early adulthood. The mean (SD) score was 46.2 (9.8) (n = 87) in group 1 versus 45.1 (8.3) (n = 94) in group 2, (P = 0.44). In contrast, the two groups differed significantly on the midlife I-S-T 2000 R cognitive scores. The mean (SD) scores obtained by participants of group 1 were 42.6 (7.4) (n = 87) versus 21.0 (5.9) (n = 94) (P<0.0001) in group 2.

### Immunolocalization of p16 in labial salivary gland tissue

**Fig 1:**
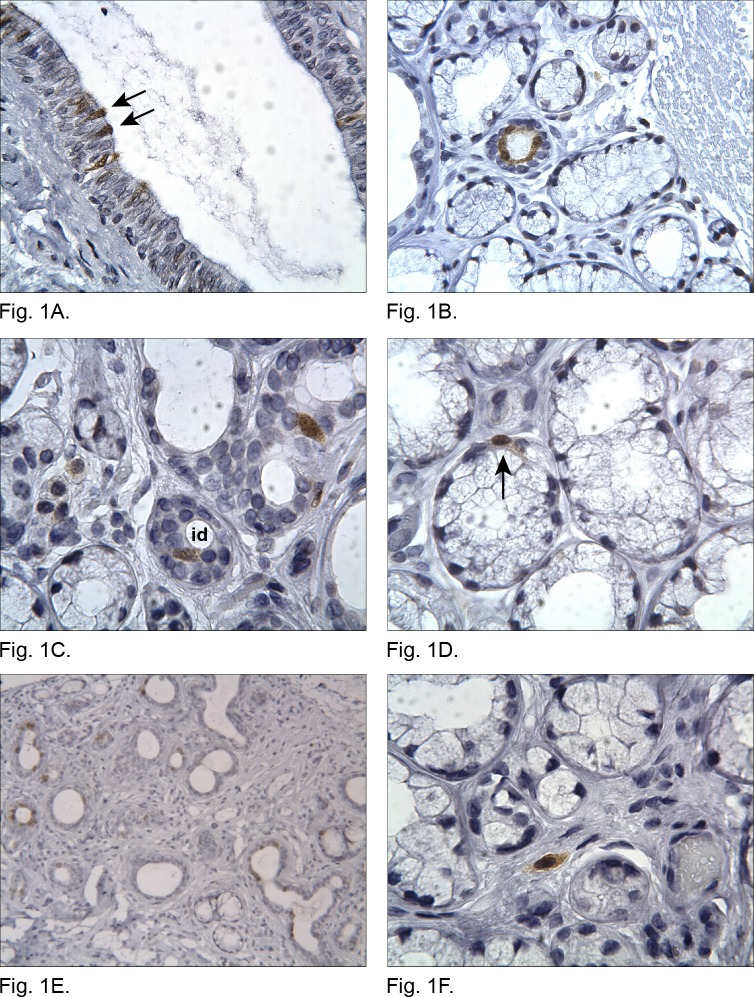
Immunolocalization of the ink4a/ARF gene product p16 in human labial salivary gland (LSG) tissue. (A) Interlobular secretory duct with several p16 positive cells located in the luminal layer (arrows) (original magnification: x250). (B) Intralobular secretory duct with luminal p16 immunoreactive cells (original magnification: x250). (C) p16 positive cell in an intercalated duct (id) (original magnification: x400) and (D) p16 positive myoepithelial cell (arrow) (original magnification: x400). (E) Atrophic area with fibrotic changes and a large proportion of secretory ducts with p16 immunoreactive cells (original magnification: x100). (F) Cells of the stroma were occasionally stained by p16 antibody (original magnification: x400).

Histopathological examination of the formalin-fixed, paraffin embedded labial salivary gland tissue samples showed that none of the samples displayed signs of malignancies. Structural degenerative changes observed in these tissue samples have been previously published and included alterations such as loss of acinar cells, fibrosis, fat infiltration, ductal dilatation, focal lymphocytic infiltrates and diffuse inflammation [24]. In order to study whether p16 is present in labial salivary gland parenchymal cells or limited to stromal cells, we analyzed the immunolocalization pattern of p16 in labial salivary gland tissue sections as shown in Fig 1A–1F) and S1 Fig. Cells, which reacted positively with the p16 antibody, displayed nuclear and cytoplasmic staining. The most intensive p16 immunoreactivity was observed in cells of inter- and intralobular secretory ducts (Fig 1A and 1B, S1B to S1H Fig). Fig 1A shows a longitudinal section of an interlobular secretory duct in which single p16 immunoreactive cells are seen in the luminal layer (arrows). Fig 1B displays a cross section of an intralobular duct with luminal p16 positive cells. In intercalated ducts single p16 positive cells were occasionally observed (Fig 1C and S1A Fig). Myoepithelial cells displayed sporadically p16 immunoreactivity (Fig 1D). Fig 1E shows an example of an atrophic lobular area with a high proportion of secretory ducts and mainly luminal cells displaying p16 immunoreactivity. Apart from the above mentioned observations, occasional endothelial and stromal cells were also stained (Fig 1F, S1I to S1K Fig). The stromal cells were distributed as single or several cells within the extracellular matrix.

### Relative p16 expression in human labial salivary gland tissue and correlation with changes in cognitive performance in late midlife

**Fig 2:**
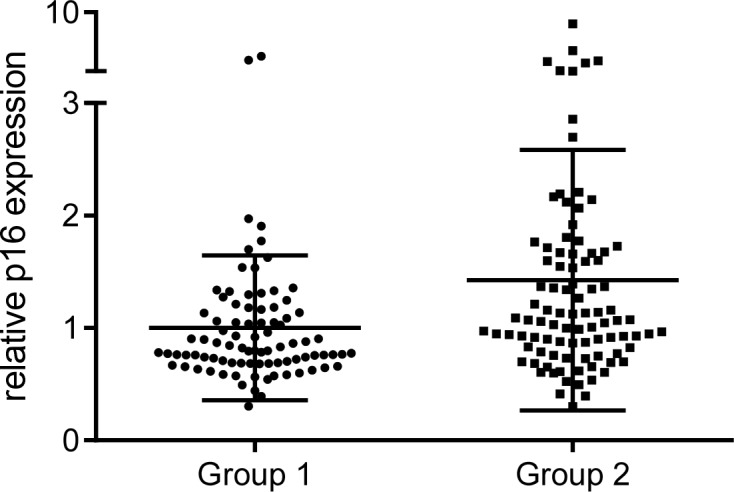
Relative p16 expression in human labial salivary glands. p16 expression values were normalized to GAPDH as reference gene. The mean relative p16 expression (horizontal lines) in group 1 was set to be 1. Group 1 refers to participants with relative increase and group 2 refers to participants with relative decrease in cognitive performance with age.

**Fig 3:**
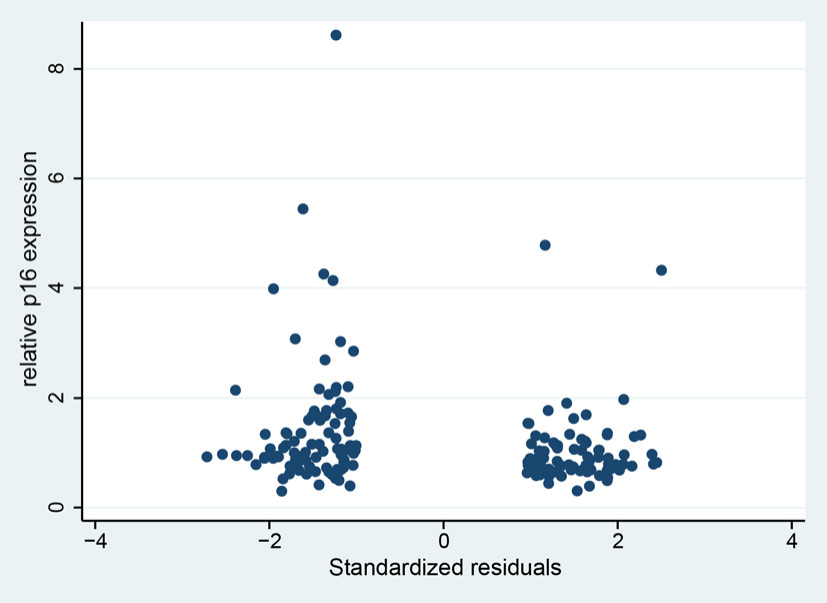
Correlation between the standardized regression residuals and the relative p16 expression values. Negative regression residuals indicate lower cognitive performance than predicted from the cognition scores obtained in early adulthood (group 2). Positive regression residuals are indicative of higher cognitive performance than predicted from early adulthood scores (group 1).

Quantitative real time PCR analysis revealed that the two groups differed significantly with respect to the mean relative expression of p16 in labial salivary glands (P = 0.003) (Fig 2), which was increased in group 2. The fold change between the means of the two groups was 1.4. Logistic regression analysis with relative p16 expression values as predictor of group classification showed that higher relative p16 expression was significantly positively associated with group 2 assignment with an OR (odds ratio) of 2.181 and 95% CI (1.265–3.761). Moreover, we found a modest but significant negative correlation between the relative p16 expression values and the standardized regression residuals (Fig 3). The Pearson’s correlation coefficient was r = -0.19 (P = 0.011, n = 181).

## Discussion

In the present study we showed that the mean expression level of the potential aging biomarker p16 in labial salivary glands differed in males with and without evidence for cognitive decline. Moreover, with higher relative expression of p16 there was an increased chance to be assigned to the group of participants, whose cognitive performance in late midlife was lower than predicted from the early adulthood scores. We also observed that p16 expression was negatively correlated with lower rate of cognitive decline as suggested by the negative correlation between the relative p16 values and the standardized regression residuals. p16 positive cells were localized both in the glandular parenchyma and in the stroma, indicating that senescent cells may accumulate in LSGs with age. p16 expression in normal LSGs may thus represent a correlate of different trajectories of cognitive aging late in midlife.

The results from previous studies on different human [13–15], as well as rodent tissues [5,12,16–19] indicate that elevated p16 expression may be related to advanced age of an organism. However, it is yet not completely understood, why p16 expression increases in parallel with age. Neither is it clear by which mechanism p16 expression in a peripheral tissue could be associated with age-related changes in cognitive performance. One assumption is that higher levels of p16 may indicate accelerated systemic aging processes, which in conjunction with decline in organismal regenerative capacity might influence the efficiency of the aging brain. It is also possible, though highly speculative, that increased p16 expression in a peripheral organ might parallel processes of cellular senescence within the central nervous system. In the brain, astrocytes, microglia or oligodendrocytes may undergo senescence and this could play a causal role in neuro-inflammatory and other pathological processes by release of pro-inflammatory cytokines or other SASP molecules [7].

Our observation that p16 is present in human salivary glands and expressed in a cell-specific pattern, is in line with results from earlier studies [14,31]. However, we found that acinar cells were virtually devoid of p16 staining, whereas both secretory duct and occasionally myoepithelial cells displayed p16 immunoreactivity. This observation is in line with p16 expression in myoepithelial and ductal, but not acinar cells of normal human salivary glands [31]. A possible explanation for the lack of p16 immunoreactivity in acinar cells could be that these cells undergo apoptosis instead of cellular senescence in response to stress stimuli, as acinar atrophy is a common age-related finding in labial salivary glands [32–35]. Another explanation could be that cellular senescence in these cells might be induced by the alternative signaling pathway that involves p14^ARF^ stabilization of p53.

Cells of intra–and interlobular secretory ducts showed the highest frequency of p16 immunoreactivity, whereas the frequency of stained intercalated duct cells and myoepithelial cells was low. The expression of p16 in secretory ducts, endothelial and myoepithelial cells as well as stromal cells suggests that they may have previously possessed mitotic activity and subsequently undergone cellular senescence, retained by an up-regulated level of p16. However, none of the markers of cellular senescence identifies all types of senescent cells or is solely specific to them.

Cellular senescence mechanisms seem to be involved in tissue repair processes as well [6]. Results from a previous study on the murine liver indicated that transition of activated hepatic stellate cells into cellular senescence was important for restraining liver fibrosis in response to injury [36]. Inhibition of senescence that impaired the proliferation arrest of these cells resulted in enhanced liver fibrosis in mice with deficient senescence response [36]. The mechanism by which senescent cells exert repair functions may involve their altered secretory phenotype, which otherwise also is thought to contribute to their role in promoting tissue aging [6]. In relation to this, we observed in an atrophic lobular area, characterized by fibrotic changes, a high proportion of intralobular secretory ducts with a relatively high number of cells displaying p16 immunoreactivity. It is thus possible that these are senescent cells involved in tissue repair processes. Another possibility is that these cells play a role in promoting fibrosis, which is one of the well characterized age-related changes in this tissue [33,34,37].

The overall expression level of p16 was relatively low in human labial salivary glands. This might be due to the fact that the samples were obtained from middle-aged and not elderly persons. Moreover, it could relate to the baseline proliferative activity of the parenchymal cells, which was very low in myoepithelial and oxyphilic cells and low in acinar, basal and intercalated duct cells [20].

In summary, our data have shown that the tumor suppressor and potential aging biomarker p16 is expressed in a specific pattern by certain cells of human salivary gland parenchyma and stroma. p16 expression levels in labial salivary glands seemed to predict cognitive decline in middle age. However, the group differences and correlation were modest, and should thus be interpreted with caution.

## Conclusions

In conclusion, labial salivary gland tissue may be suitable for studies on organismal aging processes, including cellular senescence. Our findings indicate that p16 expression in labial salivary glands is a potential peripheral correlate of changes in cognitive performance in late midlife.

## Supporting Information

S1 Fig Additional images of the p16 immunohistochemistry performed on the labial salivary gland (LSG) tissue samples. (PDF)

S1 Table Ct-values (triplicates) for p16 (CDKN2) and GAPDH. (XLSX)
