# Supplementary material for: Elevated p16ink4a Expression in Human Labial Salivary Glands as a Potential Correlate of Cognitive Aging in Late Midlife
Source: PLoS One. 2016 Mar 30;11(3):e0152612. doi: 10.1371/journal.pone.0152612 (PMC4814104; doi:10.1371/journal.pone.0152612)
Supplement: S1 Fig — (PDF) [file pone.0152612.s001.pdf]

S2 Figure

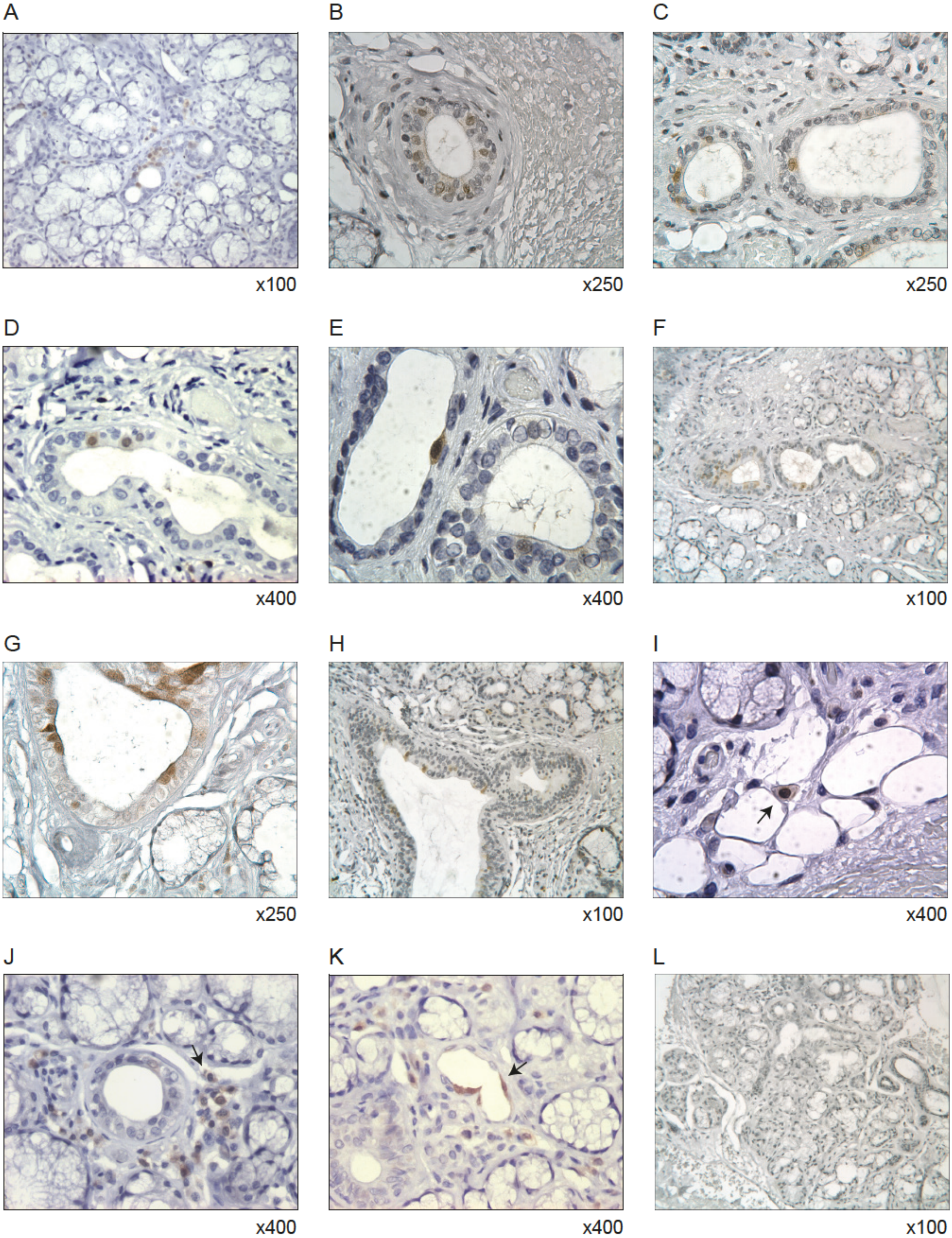

S2 Figure. A – H: Images of p16 positive cells in human labial salivary gland intercalated, intra – and interlobular secretory ducts (original magnification as indicated). I and J: Single cells of the stroma, displaying p16 immunoreactivity (arrows). K: p16 positive endothelial cells (arrow). L: Control image with primary antibody omitted.
